# A Comparative Analysis of Language Skills and Parent–Child Interactions in Monolingual and Bilingual Children Born Preterm

**DOI:** 10.3390/languages9120361

**Published:** 2024-11-25

**Authors:** Kimberly Crespo, Emma Libersky, Julie Poehlmann, Margarita Kaushanskaya

**Affiliations:** 1 Department of Speech, Language and Hearing Sciences, Boston University, Boston, MA 02215, USA; 2 Department of Communication Sciences and Disorders, University of Wisconsin-Madison, Madison, WI 53706, USA; 3 Department of Human Development and Family Studies, University of Wisconsin-Madison, Madison, WI 53706, USA

**Keywords:** preterm, language development, parent–child interactions, bilingualism

## Abstract

Children born preterm are at an elevated risk of language delays compared to children born full-term. However, there is a dearth of research investigating language outcomes in premature children exposed to more than one language. There is also limited empirical evidence linking the quantity and quality of parent input and language outcomes in premature children and the strength of these relationships in bilingual contexts remains unknown. Therefore, the current study examined language skills, parent input, conversational turns, and their associations at 16 months to 36 months in monolingual and bilingual children born preterm. Nine English-speaking monolingual parent–child dyads, and nine Spanish–English bilingual parent–child dyads participated in parent–child interactions that occurred over time in play-based contexts. Results revealed that preterm monolingual and bilingual children exhibited similar language abilities at all time points assessed. While both monolingual and bilingual dyads engaged in a comparable number of conversational turns at 16 m, monolingual mothers produced more words than bilingual mothers during play. Significant associations were observed between children’s vocabulary skills and their ability to combine words within and across most time points. Notably, relationships between parental input, conversational turns, and language skills were limited to a significant association between conversational turns at 16 m and vocabulary skills at 24 m. Together, findings indicate that bilingual children born preterm acquire language on the same timeline as monolingual children born preterm. While it is crucial that the current work be replicated in larger samples of children born preterm, the current work is the first to compare relationships between children’s language outcomes and the quantity and quality of parental input in monolingual and bilingual contexts.

## 1. Introduction

Due to remarkable biomedical advances, the survival rate of children born preterm before 37 weeks gestation has substantially improved (Stoll et al. 2015). However, differences in brain development, higher likelihoods of exposure to adverse social and environmental factors associated with premature birth, and lower mortality rates have led to an increase in the number of children at neurodevelopmental risk (e.g., Anthopolos et al. 2014; Maalouf et al. 2001; Masho et al. 2017; Peterson et al. 2002; Wheeler et al. 2018). Studies suggest that, in addition to poorer health outcomes (Escobar et al. 2005; Hibbard et al. 2010; Shapiro-Mendoza et al. 2006; Stoll et al. 2015), preterm children have higher rates of developmental delays in cognitive (e.g., Bhutta et al. 2002; Ortiz-Mantilla et al. 2008), motor (e.g., Bos et al. 2013), and social-emotional skills (e.g., Montagna and Nosarti 2016; Taylor 2020). Language development in preterm children has also been shown to be atypical (Putnick et al. 2017; van Noort-van der Spek et al. 2012; Vandormael et al. 2019; Vohr 2014). Given the strong predictive relationships between children’s language skills and their neurodevelopmental outcomes (e.g., Desmarais et al. 2008; Hoff 2013; Mitchell et al. 2006; Snowling et al. 2016), there is growing interest in identifying factors that may enhance language development in all children, including children born preterm. The quantity and quality of parental input, for example, has received significant attention due to the relevance of social communication to language development (e.g., Hoff 2006; Rowe 2012).

To date, research in this area has predominantly focused on monolingual English-speaking children. As a result, there is a dearth of research investigating language outcomes in preterm children who are exposed to multiple languages. There is also limited empirical evidence linking linguistic and interactive features in parent–child interactions with language outcomes in preterm children over time, particularly in bilingual contexts. In the current study, we conducted secondary analyses of video-recorded mother–child interactions that were collected in a longitudinal project investigating cognitive and social-emotional development in preterm children. Here, we examined the relationship between quantity and quality of parental input at 16 months, and children’s expressive language skills, at 16 months (16 m) and 36 months (36 m) corrected age in monolingual and bilingual children born preterm. By investigating the intersection of two complex developmental contexts—prematurity and bilingualism, researchers can begin to identify specific vulnerabilities and critical periods for intervention across a broader population.

### 1.1. Language Skills in Preterm Children

Marked and persistent language difficulties in preterm children are well documented (e.g., Barre et al. 2011; Brósch-Fohraheim et al. 2019; Luu et al. 2009, 2011; Putnick et al. 2017; van Noort-van der Spek et al. 2012; Vandormael et al. 2019; Vohr 2014). Studies in preschoolers suggest that preterm children may present with poorer vocabulary skills and produce language with less complex grammatical and syntactic features (e.g., Brósch-Fohraheim et al. 2019). School-aged children born preterm also present with poorer language abilities and are reported to underperform academically, on average. A recent meta-analysis found moderate to large differences in measures of language, reading, and mathematics skills in school-aged children born preterm compared to their full-term peers (e.g., McBryde et al. 2020). Moreover, Developmental Language Disorder (DLD)—a communication disorder that is not associated with a known biomedical etiology and interferes with learning, understanding, and using language (Bishop et al. 2016, 2017)—has been observed in approximately 20–30% of preterm children, compared to 3–10% in children born full-term (e.g., Mossabeb et al. 2012; Sanchez et al. 2019; but see St. Clair et al. 2019 for similar rates of DLD reported in preterm and full-term children). There is also some evidence to suggest that language difficulties in preterm children may persist through adolescence (e.g., Luu et al. 2009, 2011).

Whereas language difficulties are common, it is important to note that there is significant heterogeneity in language abilities in children born prematurely. Not all preterm children exhibit deficits in receptive (e.g., Vandormael et al. 2019), expressive (e.g., Brósch-Fohraheim et al. 2019; Soraya et al. 2012), or discourse-level language skills (e.g., Smith et al. 2014). The heterogeneity in this group may suggest that the causal relationship between preterm birth and language development is not linear. Instead, the effects of prematurity on language development may be more complex and modulated by linguistic and interactive factors in the language environment (e.g., De Leeuw et al. 2024).

Parent–child interactions and the language environment are intrinsically linked in early development as parents are often children’s first contact with essential vocabulary, grammar, and conversational norms (e.g., Weisleder and Fernald 2013). Research has consistently shown robust links between parental input and children’s language skills (e.g., Anderson et al. 2021; Hoff 2006; Rowe 2012; Romeo et al. 2018; Weisleder and Fernald 2013). A recent meta-analysis by Anderson et al. (2021) compared the magnitude of the associations between the quality and quantity of parental input and language skills. Results revealed significant moderate to strong associations between the quality (indexed by vocabulary diversity and syntactic complexity) and quantity (indexed by number of words or utterances) of parent input and children’s language development. In the present study, we focus on the quality of parent–child interactions and language skills in pre-term monolingual and bilingual children.

### 1.2. Parent–Child Interactions in Preterm Children

Given differences in language ability between children born full- and pre-term, an open question is whether preterm children may be less sensitive to parental input given their atypical neurodevelopmental trajectories. There is precedent for this hypothesis in other populations. For example, Blom et al. (2023) found that the receptive vocabulary skills of children suspected of having DLD were not related to the number of adult words derived from day-long recordings in the home. As noted by the authors, it is possible that processing, memory, and/or attention deficits associated with language delays and disorders may hamper children’s ability to use linguistic input for language learning. Only a handful of studies have investigated this question (e.g., Adams et al. 2018; Coughlan et al. 2024), and the results suggest that language outcomes and parental input may be significantly associated with preterm children. However, one limitation of this literature is that the few studies conducted on the topic have either been constrained to a single time point or to measurements of language abilities up to 30 months. Studies that go beyond 30 months have focused on parent-reported or standardized measures of children’s vocabulary skills (e.g., Soraya et al. 2012). Consequently, we have a limited understanding of how interactions with parents influence preterm children’s communication skills across different stages of early language development.

In the present study, we analyzed children’s spontaneous language skills during play at multiple time points to compute their Mean Length of Utterance in words (MLUw)—the average number of words per utterance. MLUw is a key index of linguistic productivity, offering valuable insights into children’s ability to combine words and construct longer and more complex sentences. Given our focus on bilingualism, we calculated MLU in words rather than MLU in morphemes because it is developmentally sensitive and better for comparing speakers of multiple languages (e.g., Gutiérrez-Clellen et al. 2000; Rojas and Iglesias 2013). We also examined parent-reported expressive vocabulary skills for the children. A primary aim of this work was to examine whether preterm children’s expressive language skills at 16 and 36 months (indexed via parent-reported vocabulary and MLUw derived from spontaneous speech) were related to the quantity and quality of parental input at 16 months.

The quantity of language input, often measured by the number of words spoken by caregivers, has been strongly associated with children’s vocabulary development as well as overall language abilities (e.g., Brito et al. 2020; Hoff 2006; Huttenlocher et al. 2002; Romeo et al. 2018; Rowe and Weisleder 2020). Studies have consistently reported that typically developing children exposed to a greater quantity of language demonstrate larger vocabularies and more advanced language skills than children who receive less input. In the current study, the quantity of parental input was operationalized as the number of total words (NTW) produced by mothers during play interactions. The use of NTW as a proxy for language quantity was chosen to align with prior studies that have used daylong-recordings (which cannot estimate the number of *different* words a child is exposed to) to measure language to children born preterm (e.g., Adams et al. 2018) as well as studies that have sought to index language quantity separately from the quality or interactivity of language input (e.g., Hirsh-Pasek et al. 2015).

The quality of language input has also been shown to play a crucial role in shaping neurotypical children’s language outcomes (e.g., Rowe 2012; Tamis-LeMonda et al. 2012; Tamis-LeMonda et al. 2014). There are several key metrics of language quality that are relevant to parent–child interactions. For example, caregiver lexical diversity (e.g., Hoff and Naigles 2002; Venker and Johnson 2022) and caregiver MLU (e.g., Girolametto et al. 1995; Roberts and Kaiser 2015) provide insight into the range of vocabulary and the grammatical richness of caregiver input, and research suggests that variation in these factors shape language outcomes in children with a range of language abilities. In early interactions, metrics of language quality are also often characterized by the frequency and responsiveness of conversational turns. Like other measurements of language quantity, early conversational turns have been positively associated with children’s later language outcomes (e.g., Rowe 2012; Romeo et al. 2018; Tamis-LeMonda et al. 2014).

In this investigation, we examined associations between parental input at 16 months and children’s expressive language skills at 16 m and 36 m in monolingual and bilingual preterm children. Language development and parent–child interactions undergo significant changes from 16 m to 36 m, when children begin to form simple sentences with increasing grammatical complexity (Levickis et al. 2018; Silvey et al. 2021). The dynamics in shared interactions also change during this period as a function of children’s increasing capacity to understand, speak, and take conversational turns (e.g., Levickis et al. 2018; Silvey et al. 2021). Yet, it remains unclear how the associations between parent–child interactions and children’s expressive language evolve over time in children born preterm. In the current study, we were especially interested in testing the strengths of these associations in bilingual vs. monolingual children.

### 1.3. Bilingualism Effects on Language and Parent–Child Interactions in Preterm Children

Research on the intersection of prematurity and bilingualism is an emerging field. Available studies investigating language skills in bilingual preterm children (e.g., De Leeuw et al. 2024; Sanchez et al. 2019; van Veen et al. 2019; Walch et al. 2009) suggest that preterm children who are raised in bilingual environments may have poorer language skills than preterm children who are exposed to only one language in the home.

Critically, much of the research in infants and toddlers has relied on measures that were developed and standardized on monolingual children. For example, the Bayley Scales of Infant and Toddler Development (Bayley 2006) has been the predominant instrument for measuring young children’s language skills in recent studies (e.g., De Leeuw et al. 2024; Sanchez et al. 2019; van Veen et al. 2019; Walch et al. 2009). Studies in school-aged bilingual children with a history of preterm birth have used standardized measures of vocabulary in each language (e.g., Baralt and Mahoney 2020). Although the Bayley and language-specific vocabulary measures are psychometrically robust tools, they were developed and normed on monolingual children. Monolingual-standardized language assessments are unsuitable for bilingual children and may underestimate their abilities (e.g., Rose et al. 2022). In addition, measurements primarily normed on Western monolingual English-speaking populations may not account for cultural differences in child-rearing practices and communication styles and expectations that may characterize non-Western and immigrant families. Play-based parent–child interactions offer a more holistic and culturally sensitive approach to assessing the language abilities of culturally and linguistically diverse children (e.g., Cote and Bornstein 2009; Ebert 2020). Therefore, in the present study, we examined bilingual preterm children’s language skills via naturalistic play interactions with their parents.

A critical factor to consider when examining the effects of bilingualism on language skills in preterm children is the distributed nature of bilingual language exposure. Distributed exposure has been linked to smaller language-specific vocabularies (e.g., Hoff et al. 2012; Hoff and Ribot 2017; Pham and Kohnert 2014) and slower early grammatical development (e.g., Blom 2010; Hoff et al. 2012) compared to monolingual norms. In our analyses, we focused on bilingual children’s *conceptual vocabulary*, which indexes the number of concepts children know rather than the number of language-specific words. In this approach, concepts that can be labeled in both languages are counted only once. Conceptual vocabulary has been shown to provide a more comprehensive measure of bilingual children’s linguistic competence than vocabulary size in each language separately (e.g., Bedore and Peña 2008; Gross et al. 2014). Indeed, conceptual vocabulary is particularly informative because bilingual children often have gaps in their vocabulary in each language due to varied contexts of language exposure and use.

A key question in the current work was whether differences would be observed in the quantity and quality of parent–child interactions between monolingual and bilingual mother–child dyads. There are a complex interplay of cultural, linguistic, and parental factors that shape parent–child interactions, particularly in the context of neurodevelopment disorders (e.g., Britsch and Iverson 2024; Castagna et al. 2024; Hoff and Shanks 2024). Compared to monolingual parents, recent research suggests that bilingual parents may be less responsive to bids for communication from children with developmental language impairments in their native language (Smith et al. 2020). Smith et al. (2020) additionally found that the use of non-native English did not appear to adversely affect how often, or how quickly, bilingual parents responded to their children’s verbal communication bids. However, there was substantial within-group variability in this study, and the authors argue that bilingual parental responsiveness to child communication acts may have been more closely linked to their proficiency in English rather than bilingualism per se. Other work has found that monolingual and bilingual parents may not differ in their interactions or the amount of language used with their children (e.g., Gampe et al. 2020).

In addition to differences across parent groups, differences in child characteristics between monolinguals and bilinguals may shape parent–child interactions and consequently children’s language skills. For example, studies show that bilingual and monolingual children weigh verbal and social communication cues differently (e.g., Brojde et al. 2012; Gangopadhyay and Kaushanskaya 2020; Verhagen et al. 2017; Yow and Markman 2015). In some cases, bilingual children rely more on social cues to learn, such as pointing and eye gaze, than their monolingual peers (e.g., Brojde et al. 2012; Verhagen et al. 2017). Bilingual children may also be better than monolingual children at adapting their communication to meet the needs of their communication partners (e.g., Gampe et al. 2019; Wermelinger et al. 2017; Yow and Markman 2016), and at integrating communicative cues to interpret their partner’s intended meaning (Yow and Markman 2011, 2015). Taken together, heightened sensitivity to the communicative context and to interaction partners may enhance the learning potential of parent–child interactions for dual language learners.

### 1.4. Current Study and Hypotheses

In the present study, we examined relations between parental input, operationalized as a total number of words and conversational turns at 16 months, and children’s expressive language skills, operationalized as parent-reported expressive vocabulary and MLUw in spontaneous speech (calculated from recorded parent–child interactions), at 16 m and 36 m corrected age in monolingual and bilingual children born preterm. In addition, we report the use of English, Spanish, and language mixing during parent–child interactions for bilingual mothers and children at 16 m and 36 m.

We also conducted secondary analyses linking the quantity of parent input, operationalized as a total number of words, at 36 m, with children’s expressive language at 36 m, to examine whether there would be similarly strong relationships between parent and child data at earlier (16 m) and later (36 m) time points. We did not examine conversational turns at 36 m because conversational turns are typically considered an early predictor of language growth (e.g., Hirsh-Pasek et al. 2015; Smith et al. 2018). We also conducted a secondary analysis examining the link between input factors at 16 m and parent-reported expressive vocabulary at 24 m. Unfortunately, parent–child interaction data were not available for 24 m, but the availability of parent-reported expressive vocabulary enables us to consider the connection between parent input and children’s language outcome at a more proximal time point. Given the dearth of studies conducted in bilingual preterm children, it was challenging to posit firm hypotheses about relationships between bilingualism, parent input, conversational turns, and language skills. Here, we considered multiple exploratory hypotheses.

The evidence is clear that while learning two languages may affect the trajectory of language acquisition, it does not lead to or exacerbate language delays or neurodevelopmental disorders (e.g., Höhle et al. 2020; Uljarević et al. 2016). However, one hypothesis is that reduced language-specific input may protract the development of early language skills in pre-term children, who are particularly neurobiologically vulnerable. If so, then we anticipate that bilingual children will obtain smaller vocabulary size and MLUw and combine fewer words than monolingual children during parent–child interactions at one or more time points. An alternative hypothesis is that enhanced perceptual and cognitive flexibility may serve as a protective factor for bilingual preterm children. Indeed, some studies report that bilingualism may confer cognitive advantages in preterm children (e.g., Baralt and Mahoney 2020; Gillenson et al. 2023). However, others do not (e.g., Loe and Feldman 2016; van Veen et al. 2019). If preterm bilingual children have enhanced cognitive skills, then their vocabulary skills and MLUw may be comparable to their monolingual peers despite reduced language-specific exposure across time. A third hypothesis is that increased cognitive flexibility may bolster language skills in bilingual preterm children compared to monolingual preterm children. If so, then we anticipate that bilingual children will obtain larger vocabulary size and MLUw and produce more complex sentences than monolingual children during parent–child interactions at one or more time points.

Our hypotheses related to parent–child interactions and its links to children’s expressive language skills over time follow the same logic above. One hypothesis is that monolingual and bilingual parents will not differ in NTW or in the number of conversational turns taken with their preterm children. We may also observe that the relationship between NTW, conversational turns, and language skills is similar in monolingual and bilingual preterm children at one or more time points. An alternative hypothesis is that bilingual parents of preterm children may produce fewer NTW and take fewer conversational turns during play; and the relationship between NTW, conversational turns, and language skills may also be weaker than relationships observed in preterm monolingual dyads. A third hypothesis is that bilingual parents may produce more input and take more turns than monolingual parents; and the relationship between NTW, conversational turns, and language skills may be more robust in bilingual dyads than in monolingual dyads.

## 2. Materials and Methods

We conducted analyses of video-recorded mother–child interactions that were originally collected for a larger longitudinal project (Poehlmann-Tynan et al. 2015; Poehlmann et al. 2011) via the Parent–Child Early Relational Assessment (P-CERA; Clark 1999). The goal of the parent project was to investigate individual and family predictors of developmental competence in preterm children from birth to age 6 years, emphasizing the emergence of self-regulation and attachment relationships. Study enrollment took place soon after discharge from the neonatal intensive care unit (NICU). Information about neonatal health risks (e.g., birth weight, APGAR scores, ventilation) was collected via a review of medical records. Demographic information was collected via a questionnaire at NICU discharge. Here, we used information from demographic questionnaires and language surveys to characterize our sample.

### 2.1. Participants

Participants were recruited in the United States, in Madison- and Milwaukee-area hospitals in Wisconsin. Nine Spanish–English bilingual children were matched to 9 monolingual children on gender, gestational age, socioeconomic status (proxied by mother’s years of education), birth weight, one- and five-minute APGAR scores, and 16 m Bayley Mental Development Index score (Bayley 1993). Mother’s years of education were used to proxy socioeconomic status in line with other studies of language acquisition (e.g., Barak et al. 2022; Hoff et al. 2020; Maguire et al. 2018). It correlates with more complex composite measures of socioeconomic status, yet it is simple to measure and tends to be more stable than occupation or income (Bornstein et al. 2014; Hoffman 2014). Because language development was not a focus of the original study, information on children’s language backgrounds was limited. However, English proficiency for a participating caregiver was an inclusionary criterion because the study team spoke English and the questionnaires were in English. Mothers’ English vocabulary knowledge was assessed via the Peabody Picture Vocabulary Test, Third Edition; see Table 1), but mothers did not report whether they spoke English as a first or second language. Parents reported that bilingual children were exposed to both Spanish and English, but the extent of their exposure to each language is unknown. See Table 1 for participant characteristics.

We focused on the visits that took place at 16 m, 24 m, and 36 m corrected age. Corrected age was calculated by subtracting the number of days born preterm from a child’s chronological age (Committee on Fetus and Newborn 2004). Nine bilingual children and 9 monolingual children participated in the 16 m and 24 m visits, and 6 bilingual children and 8 monolingual children participated in the 36 m visit. We used pairwise deletion to account for missing data.

### 2.2. Procedure

During their visits, families completed a battery of questionnaires and assessments, as well as experimental and observational measures in a university laboratory setting.

#### 2.2.1. Language Development Survey

Parents completed the Language Development Survey (LDS; Rescorla 1989) as part of the larger Child Behavior Checklist (Achenbach and Rescorla 2000) during each visit. The LDS is a language development survey that includes questions about language development risk (e.g., Are you worried about your child’s language development? Has anyone in your family been slow to talk?). The LDS also includes questions about exposure to languages other than English, and whether a child is combining words. The LDS provides a vocabulary checklist which parents are instructed to fill out. The exact instructions are as follows: “Please circle each word that your child says SPONTANEOUSLY (not just imitates or understands). If your child says non-English versions of words on the list, circle the English word and write the first letter of the language (e.g., S for Spanish). Please include words even if they are not pronounced clearly or are in “baby talk” (for example: “baba” for bottle)”. Monolingual and bilingual families filled out identical survey forms.

#### 2.2.2. Parent–Child Interaction Activity

Interactions took place in a university lab setting in Madison, Wisconsin, USA. Because other activities during 16- and 36-month visits required the use of specific room setups (e.g., observation rooms with one-way windows) and involved an extensive battery of tests, in-home data collection was not possible. Experimenters spoke English with the families, instructing them to play with their child “as they normally would” for 15 min before leaving the room. Mother–child dyads were provided developmentally appropriate toys and books, which families could interact with (or not) as they chose.

#### 2.2.3. Transcription and Coding

Interactions were video recorded and transcribed offline. Video recordings of mother-child interactions were digitized using HandBrake 1.6.0 (The HandBrake Team 2022). Research assistants watched the videos in Express Scribe (NCH Software 2019) and transcribed them using Systematic Analysis of Language Transcripts (SALT; Miller and Iglesias 2017) software. Videos were transcribed from the moment the experimenter left the room to the moment the experimenter returned to the room. Research assistants transcribed spoken language according to SALT conventions; children’s productions were used to calculate MLUw and mothers’ productions were used to compute NTW. Bilingual Spanish–English research assistants transcribed the interactions of bilingual dyads. Each transcript was fully transcribed and then a second research assistant watched the video and made any necessary corrections to the transcript.

Conversational turns, coded at 16 m only, were operationalized as either a verbal or nonverbal utterance from one partner that was followed by a verbal or nonverbal utterance from the other partner within five seconds. Gestures, both representational and deictic, and vocalizations were coded to assess non-verbal turn-taking (as both verbal and non-verbal turns are predictive of language growth, e.g., Hirsh-Pasek et al. 2015; Smith et al. 2018). This code is similar to the adult–child conversational turn-taking measure calculated by the LENA Pro processing system (see Romeo et al. 2018 for an example of its use), but it accounts for nonverbal turns (see Hirsh-Pasek et al. 2015; Smith et al. 2018 for coding schemes that included non-verbal turns). Finally, interactions were coded for language of utterance in order to characterize the language use of the bilingual dyads. Each utterance was coded as English, Spanish, codeswitched (if both English and Spanish were used), or neutral. Utterances were coded as neutral if they were non-linguistic (i.e., gestures or vocalizations) or language could not be determined (e.g., a single word utterance that could exist in either language, like “no”).

To assess coder reliability, 10% of the videos were double-transcribed and double-checked by a second set of coders. Intra-class correlation coefficients (ICCs) were calculated for each measure that was analyzed, comparing values from the original and double-coded transcripts. ICC values range between 0 and 1, where values below 0.5 indicate poor reliability; values between 0.5 and 0.75 suggest moderate reliability; between 0.75 and 0.9 indicates good reliability; and above 0.9 suggests excellent reliability. For 16 m transcriptions, reliability was 0.90 for the mother’s NTW; 0.82 for the child’s MLUw; and 0.89 for conversational turns. For 36 m transcriptions, reliability was 0.90 for the mother’s NTW and 0.99 for the child’s MLUw.

### 2.3. Analysis

In this study, the distribution of the data was markedly non-normal as indicated by skewness and kurtosis metrics. Transformations such as log, square-root log, and Box–Cox for right-skewed data did not sufficiently normalize the data. Therefore, non-parametric statistics were deemed to be the most appropriate analytical choice because they are robust against distribution violations.

All computations were conducted in RStudio, version 2022.7.1.554 (R Studio Team 2022). We conducted the Wilcoxon rank sum test with continuity correction via the *stats* package (R Core Team 2022) to examine differences between monolingual and bilingual preterm dyads. A continuity correction is an adjustment that is made when a discrete distribution is approximated by a continuous distribution. The Wilcoxon rank sum test, also known as the Mann–Whitney U test, is a non-parametric test often used in place of the two-sample t-test when the normality assumption is not met. The Wilcoxon rank sum test is used to compare the median of two groups, or to test whether the distributions of two groups are significantly different from each other. This test also returns a “difference in location” measure. This measure does not estimate the difference in medians; instead, it calculates the median of the differences between paired samples, where each pair consists of one observation from monolingual data and one from bilingual data. In essence, this involves computing the difference for each possible pair and then determining the median of these differences. Here, we also used the *rstatix* package (Kassambara 2021) to compute the Wilcoxon effect size (*r*), which ranges from 0 to 1. Common interpretations of values for *r* are 0.10–0.29 = small effect; 0.30–0.49 = moderate effect; and 0.50–1.00 = large effect.

Spearman’s rank correlations were computed using the Hmisc package (Harrell 2024) to examine associations between parent input, conversational turns, and children’s language skills, both within and across time points. Spearman correlations were first conducted on the entire sample, and then by language group separately. We conducted correlations for the entire sample of children because, given the small sample size, pooling the children together provided more statistical power to examine the relationships between the variables of interest. Conducting correlations on the whole sample first also allowed us to identify general trends that may apply to preterm children’s language development. Re-running the correlations separately by language group allowed us to analyze whether differences in bilingual experiences alter the overarching associations observed between the variables in the whole sample.

Spearman’s test can be used to analyze ordinal level, as well as continuous level data in small sample sizes because it uses ranks instead of assumptions of normality. Spearman’s rho (*r_s_*) correlation coefficient is typically interpreted as: 0.10–0.40 = weak correlation; 0.40–0.70 = moderate correlation; and 0.70–1.00 = strong correlation. Corrections for multiple comparisons were not conducted given the exploratory nature of the study and precedent set in the literature in similar studies with small samples of specialized participant populations (e.g., Girolamo and Rice 2022; Lorang and Sterling 2021; Mason-Apps et al. 2018).

## 3. Results

### 3.1. Wilcoxon Rank Sum Tests Comparing Monolinguals and Bilinguals

#### 3.1.1. Vocabulary

**Figure 1.**
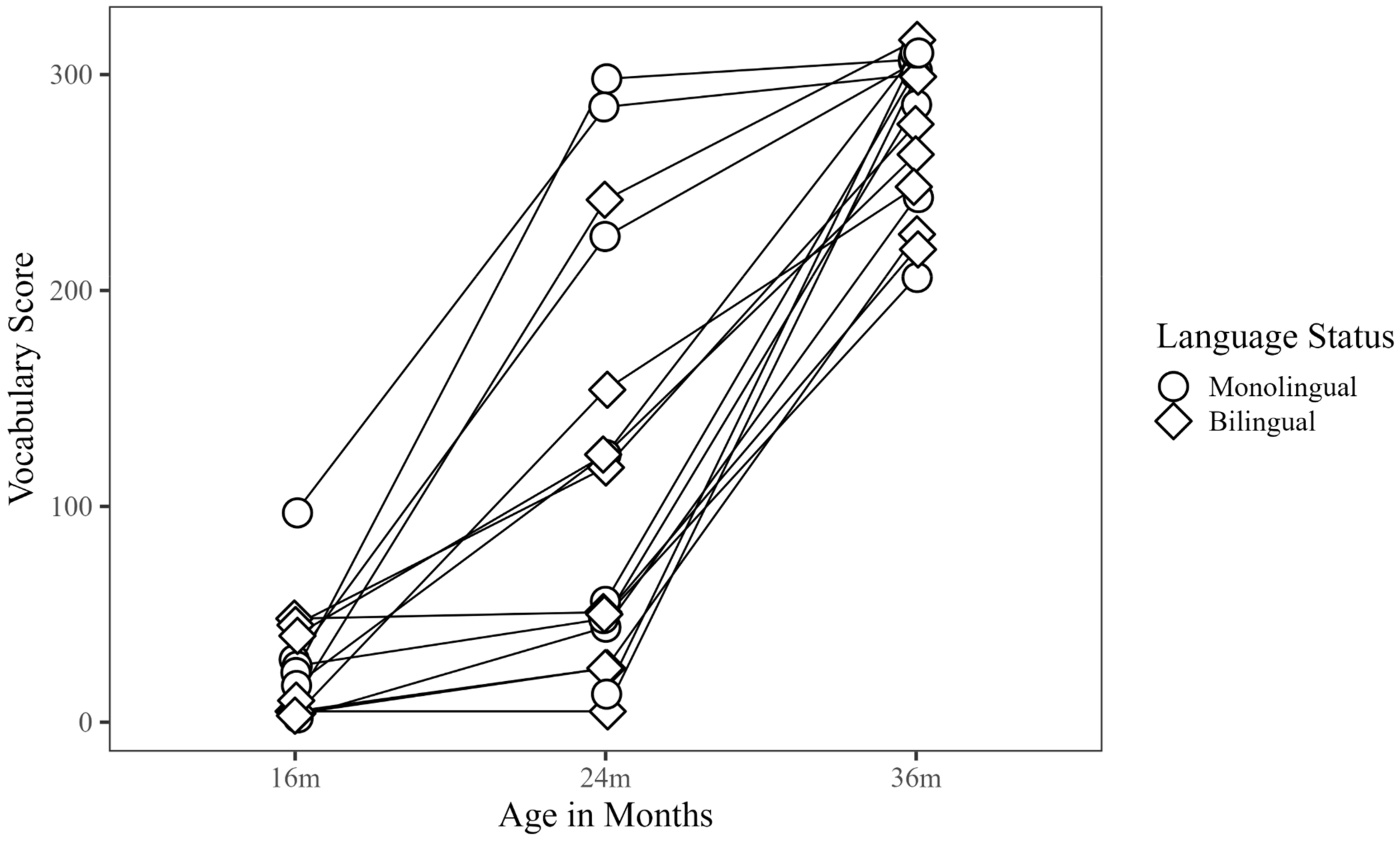
Vocabulary skills in monolingual and bilingual children, 16 m–36 m.

Patterns in the raw data revealed that preterm monolingual children produced more words than bilingual children at both time points. However, results from Wilcoxon rank sum tests revealed that group differences between preterm monolingual and bilingual children were not statistically significant at 16 m (*W* = 20, *p* = 0.65, *r* = 0.15), 24 m (*W* = 33.50, *p* = 0.57, *r* = 0.15) or 36 m (*W* = 20, *p* = 0.24, *r* = 0.31) (Figure 1). See Table 2 for vocabulary descriptive statistics by language group.

#### 3.1.2. Mother–Child Interactions

At 16 m, on average, bilingual mothers produced a greater proportion of utterances in English (*M* = 0.62, *SD* = 0.42; *Range* = 0.05–0.98) than in Spanish (*M* = 0.28, *SD* = 0.41; *Range* = 0.00–0.89) and produced few utterances that mixed both languages (*M* = 0.02, *SD* = 0.02; *Range* = 0.00–0.07). At 36 m, bilingual mothers produced an even greater proportion of English utterances (*M* = 0.77, *SD* = 0.35; *Range* = 0.07–0.96) than Spanish utterances (*M* = 0.12, *SD* = 0.30; *Range* = 0.00–0.74), and few utterances that mixed both languages (*M* = 0.02, *SD* = 0.04; *Range* = 0.00–0.10).

At 16 m, preterm bilingual children also produced a greater proportion of utterances in English (*M* = 0.05, *SD* = 0.14; *Range* = 0.00–0.42) than in Spanish (*M*= 0.01, *SD* = 0.02; *Range* = 0.00–0.07). Bilingual children in this sample did not produce utterances that mixed both languages at 16 m. At 36 m, the proportion of English utterances (*M* = 0.52, *SD* = 0.23; *Range* = 0.10–0.73) compared to Spanish utterances (*M* = 0.08, *SD* = 0.18; *Range* = 0.00–0.45) increased. Bilingual children also produced few utterances that mixed both languages at 36 m (*M* = 0.01, *SD* = 0.01; *Range* = 0.00–0.04).

**Figure 2.**
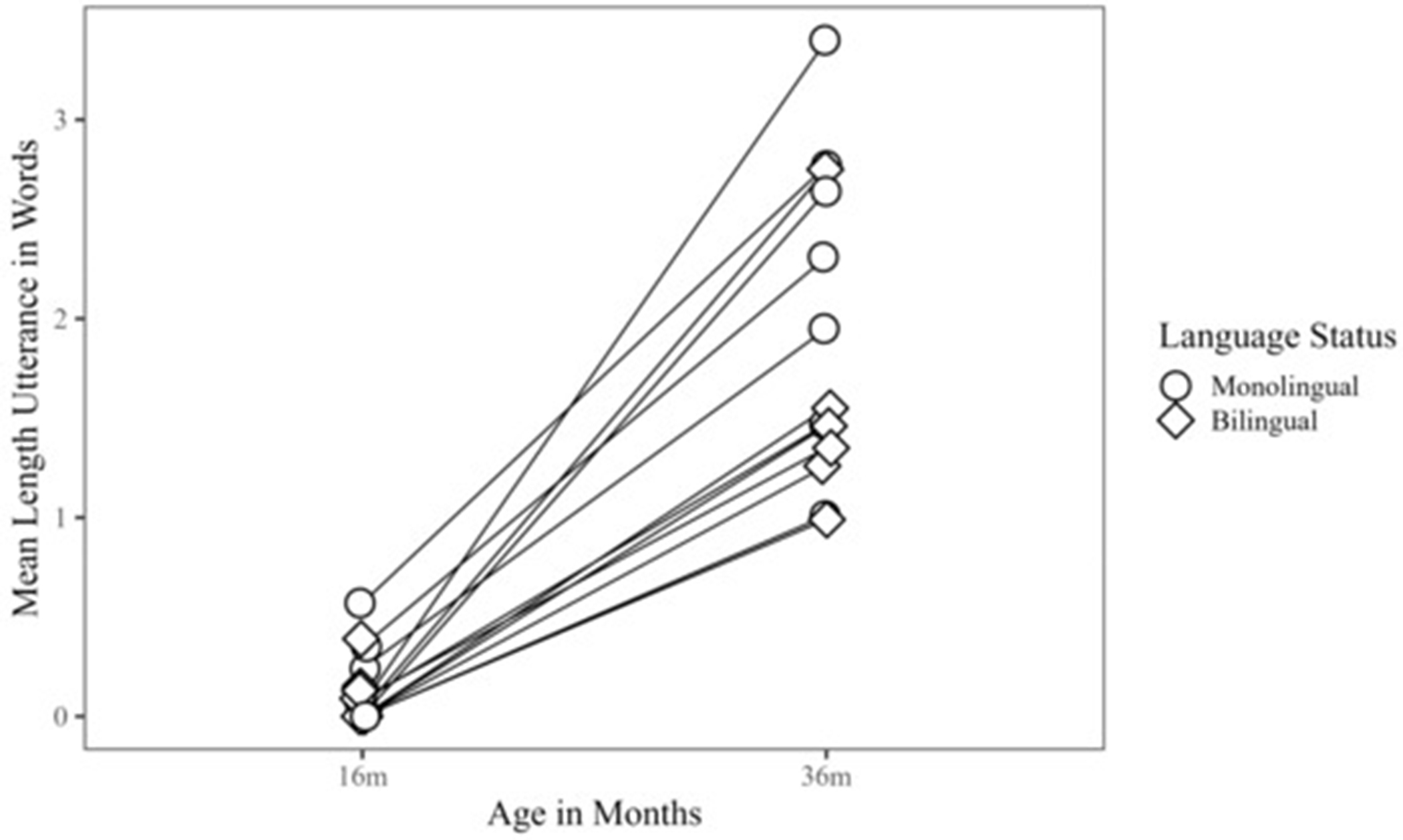
Mean Length of Utterance in Words in Monolingual and Bilingual Children, 16 m–36 m.

Patterns in the raw data revealed that preterm monolingual children combined more words as measured by MLUw than bilingual children at both time points (Figure 2). However, Wilcoxon rank sum tests revealed that group differences in MLUw between monolingual and bilingual preterm children were not statistically significant at 16 m (*W* = 46.50, *p* = 0.62, *r* = 0.13) or 36 m (*W* = 32.00, *p* = 0.13, *r* = 0.44). While not significant, group difference at 36 m yielded a moderate effect size. Based on sample estimates, the difference in location between the two distributions at 36 m was 0.71, which may reflect a meaningful difference between MLUw in monolingual preterm children compared to bilingual preterm children. See Table 2 for descriptive statistics of parent–child interaction variables by language group.

**Figure 3.**
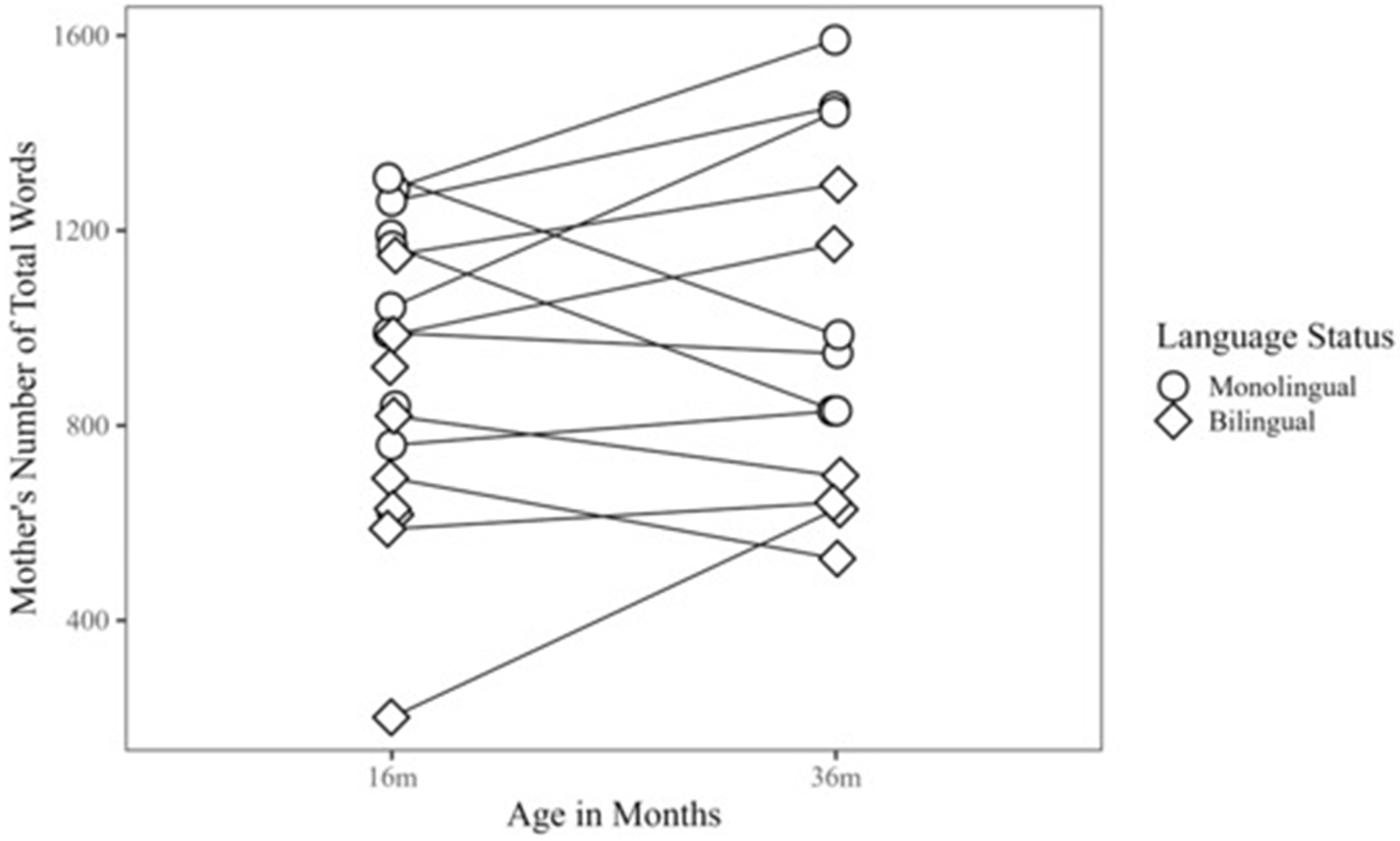
Mother’s number of total words for monolingual and bilingual children, 16 m–36 m.

When preterm children were 16 m, monolingual mothers (*M* = 1093.89, *SD* = 198.18; *Range* = 760.00–1308.00) were significantly more talkative than bilingual mothers (*M* = 733.56, *SD* = 276.04; *Range* = 201.00–1149.00), producing a greater NTW during play (*W* = 72.00, *p* < 0.01, *r* = 0.66) (Figure 3). The confidence interval for NTW was wide due to our small sample size, but we can conservatively estimate that the median number of words produced by monolingual mothers was at least 123 more words than the median number of words produced by bilingual mothers. Moreover, based on sample estimates, the difference in location between the two distributions was 360.99 words, reflecting the relatively large difference between the number of words produced by monolingual vs. bilingual mothers at 16 m.

At 36 m, monolingual mothers (*M* = 1154.57, *SD* = 327.90; *Range* = 830.00–1591.00) were only marginally more talkative than bilingual mothers (*M* = 826.67, *SD* = 321.82; *Range* = 527.00–1294.00; *W* = 34.00, *p* = 0.07, *r* = 0.52). While not statistically significant, based on sample estimates, the difference in location between the two distributions was 299.18 words. The observed large effect size may reflect a meaningful difference between the number of words produced by monolingual vs. bilingual mothers at 36 m.

Patterns in the raw data revealed that bilingual dyads (*M* = 51.56, *SD* = 28.43; *Range* = 19.00–86.00) engaged in more conversational turns than monolingual dyads (*M* = 45.33, *SD* = 23.31; *Range* = 14.00–80.00) at 16 m. However, statistical analyses revealed that this difference in the number of conversational turns was not statistically significant (*W* = 46.50, *p* = 0.62, *r* = 0.13).

### 3.2. Spearman Correlations Testing Relationships Between Parent Input, Conversational Turns, and Language Outcomes

Collapsing across language groups, Spearman correlations revealed significant associations between vocabulary skills and MLUw at 16 m (*r_s_* = 0.64, *p* < 0.01) and 36 m (*r_s_* = 0.73, *p* < 0.05). This finding suggests that monolingual and bilingual children with robust vocabulary skills combined more words than children with weaker vocabulary skills at 16 m and 36 m. Other associations between parent input, conversational turns, and children’s language skills at 16 m and 36 m were not significant (*ps* < 0.05).

Significant associations across time points were also observed. A significant correlation between MLUw at 16 m and vocabulary skills at 24 m was observed (*r_s_* = 0.62, *p* < 0.05). Preterm children who were combining more words at 16 m had a larger vocabulary size at 24 m than preterm children who were combining fewer words (i.e., smaller MLUw). Results also revealed a significant association between conversational turns at 16 m and vocabulary size at 24 m (*r_s_* = 0.66, *p* < 0.05). Children who engaged in more conversational turns with their mothers at 16 m were reported to have a larger productive vocabulary at 24 m than children who took fewer conversational turns during parent–child interactions. Productive vocabulary at 24 m significantly correlated with MLUw at 36 m, (*r_s_* = 0.62, *p* < 0.05), suggesting that children with robust vocabulary skills at 24 m combined more words when generating sentences at 36 m than children with weaker vocabulary skills. Results also revealed a significant correlation between mother’s NTW at 16 m and 36 m (*r_s_* = 0.85, *p* < 0.001); mothers who exhibited high word production during play-based interactions at 16 m were also found to demonstrate greater linguistic productivity during interactions at 36 m.

Examining relationships among the variables of interest in each preterm group separately revealed different patterns of significance. For monolingual preterm children, only MLUw and vocabulary skills at 16 m were significantly correlated (*r_s_* = 0.66, *p* < 0.05). For bilingual preterm children, results revealed a significant correlation between the number of conversational turns at 16 m and vocabulary skills at 24 m (*r_s_* = 0.79, *p* < 0.01). Vocabulary skills at 24 m were also significantly associated with bilingual children’s MLUw at 36 m (*r_s_* = 0.75, *p* < 0.01). All other relationships between parental input, conversational turns, and language skills in monolingual and bilingual children within and across time points were not significant. See Tables 3–5 for correlation matrices.

## 4. Discussion

In the current study, we investigated relations among parental input, conversational turns, and expressive language skills at 16 m, 24 m, and 36 m in preterm monolingual and bilingual children. Results revealed that preterm monolingual and bilingual children exhibited similar language abilities at all time points assessed. While both monolingual and bilingual dyads engaged in a comparable number of conversational turns at 16 m, monolingual mothers produced more words than bilingual mothers during play. Correlation analyses revealed significant associations between children’s vocabulary skills and their ability to combine words (i.e., MLUw) within and across most time points, suggesting that preterm children with larger vocabularies tended to produce more complex sentences. Notably, relationships between parental input, conversational turns, and language skills were limited to a significant association between conversational turns at 16 m and vocabulary skills at 24 m. Separate analyses of monolingual and bilingual groups revealed a significant association between conversational turns and vocabulary skills in bilingual preterm children. However, we found no evidence of such an association in monolingual preterm children.

Taken together, our findings suggest that, despite differences in caregiver input, bilingual and monolingual pre-term children in our study developed comparable vocabulary skills and sentence complexity from 16 m to 36 m. These results indicate that bilingual preterm children may acquire language on the same timeline as monolingual preterm children. Early language environments of monolingual and bilingual preterm children may be characterized by different patterns of linguistic exposure, both in terms of quantity and quality of caregiver input. While it is crucial that the current work be replicated in larger samples, the present study is the first investigation of the relationships between the quantity and quality of parental input and language outcomes in bilingual vs. monolingual children born preterm.

### Outcomes in Monolinguals and Bilinguals

In this study, we were interested in bilingual children’s broad language ability, and not in their language-specific skills. The use of conceptual vocabulary that aggregates children’s knowledge of concepts across both languages is well suited to this purpose because it levels the playing field between bilingual and monolingual children (e.g., Bedore and Peña 2008; Gross et al. 2014). Results revealed that monolingual and bilingual preterm children presented with similar vocabulary skills at 16 m, 24 m, and 36 m. Groups also demonstrated a similar ability to combine words at 16 m and 36 m. These results are corroborated by the significant overlap of individual scores between groups in Figures 1–3. Moreover, the small effect sizes associated with group differences in our sample provide support for the validity of prior claims regarding the absence of differences in language abilities between the two groups (e.g., Baralt and Mahoney 2020). This outcome is particularly notable given that the bilingual dyads engaged with both languages during play, and that bilingual children were exposed to input distributed across two languages. This finding aligns with emerging evidence indicating that distributed input does not impede language acquisition in neurotypical children (e.g., Höhle et al. 2020) nor exacerbate language delays in children with neurodevelopmental vulnerabilities (e.g., Uljarević et al. 2016).

However, our null effects of language group membership contrast with studies that have reported poorer language outcomes in bilingual preterm children (e.g., van Veen et al. 2019; Walch et al. 2009). One possibility is that methodological differences between the current study and previous reports underlie the discrepant findings. Specifically, the present study utilized conceptual vocabulary and MLUw computed from parent–child interactions, which are robust measures for assessing language abilities in bilingual children. In contrast, prior studies have predominantly relied on measures that were developed and standardized for monolingual learners. The use of monolingual assessment tools may have underestimated bilingual preterm children’s abilities in previous work (e.g., Bedore and Peña 2008; Rose et al. 2022). It is also possible that the small sample sizes in the current study contributed to the absence of significant group effects.

We did observe a wide range of English and Spanish used by bilingual mothers with their preterm children during parent–child interactions. This finding is consistent with the heterogeneity of dual language input practices observed in mothers with neurotypical bilingual children (e.g., Luo et al. 2020). From 16 m to 36 m, our sample of bilingual mothers increased their use of English with their preterm child, which is also consistent with longitudinal reports in typically developing bilingual mother-child interactions (e.g., Hammer et al. 2009; Welsh and Hoff 2021). A limitation of our study was that we did not have information about bilingual parents’ language use and language preferences. Another limitation was that, to maintain the integrity of the larger protocol, interactions were recorded in a laboratory setting instead of the home environment. Parent–child interactions captured in the home environment may have yielded a distinct pattern of results for our bilingual dyads, who may have felt more comfortable communicating in Spanish at home. In-home data collection may yield a more valid representation of language use in bilingual families (Kremin et al. 2022). That said, there is limited work comparing in-lab and in-home data collection directly (e.g., Manning et al. 2020, who compared monolingual samples across contexts) and the impact of the environment on bilingual language use remains unclear. This relates to a larger issue of bilingual assessment and measurement tools, which tend to be under-researched and less well-validated than their monolingual equivalents (see Arias and Friberg 2017; De Lamo White and Jin 2011 for review). Future research is needed to better document the language profiles of bilingual caregivers of preterm children and to examine how context, cultural values, education backgrounds, and societal pressures may influence caregiver ’s increasing language use over time.

In our study, group differences did emerge in parent input data, such that monolingual mothers used more language overall than bilingual mothers during play. Previous studies have reported similar findings in neurotypical children and have linked differences between Spanish-speaking and European American mother–child conversations to distinct cultural values associated with language practices in the two groups (e.g., Hoff and Shanks 2024; Greenfield et al. 2006). It is plausible that similar cultural constraints may have modulated the amount of input bilingual mothers produced in the present study. Another consideration is that bilingual mothers in our sample used more English than Spanish, and for some, English may have been their less dominant language, decreasing the quantity of language produced. Interactions between bilingual dyads were likely influenced by the setting and language context of the interactions. Interactions took place at an English-speaking university, and experimenters communicated with families entirely in English (including when giving the instruction to “play as they normally would”). As a result, bilingual dyads may have been in a “monolingual English mode” (Grosjean 1998, 2001) during the play interaction.

Despite quantitative differences, bilingual and monolingual mothers equally engaged in conversational turns with their preterm child at 16 m, consistent with findings in typically developing children (e.g., Gampe et al. 2020). This finding is also consistent with work in children with other neurodevelopmental disorders (i.e., autism; Smith et al. 2020). During English-language interactions, Smith et al. (2020) found that bilingual parents were responsive towards their children, on par with rates shown by English-speaking monolingual parents (Smith et al. 2020). Future research is needed to further elucidate how cultural and linguistic factors influence the quantity and quality of language input provided by bilingual versus monolingual parents of children born prematurely.

One unexpected finding was the weak relations between parental input, conversational turns, and language outcomes within and across time points. This pattern is incongruent with significant moderate and large effect sizes that characterize the associations between parental input and child language outcomes in neurotypical children (Anderson et al. 2021). However, in their meta-analysis, Anderson et al. (2021) also reported significant between-study heterogeneity, finding that the strength of the effects differed across development and measurements. Our findings are therefore not inconsistent with the variability of these associations reported in the literature (e.g., Anderson et al. 2021). However, given our sample size, it is plausible that even large effect sizes associated with relationships between these factors may have been insufficient to achieve statistical significance. We note that, while our sample size is small, it is relatively robust for a naturalistic language study with a highly specific population of bilingual preterm children.

Alternatively, neurodevelopmental vulnerabilities associated with premature birth may have weakened associations between parental input, conversational turns, and vocabulary growth. Notably, we observed a significant relationship between conversational turns at 16 m and vocabulary size at 24 m, consistent with theoretical frameworks that emphasize social interaction as a key component of language acquisition (e.g., Donnelly and Kidd 2021). Further separate analyses of language groups revealed that the statistical significance of this relationship held in bilingual children, but not in monolingual children. It is crucial to interpret these findings with caution. The null finding regarding an association between conversational turns and vocabulary skills in monolingual children should not be construed as evidence of the absence of this relationship. Several factors could have contributed to this pattern of results, such as sample size restrictions, which may have resulted in only being able to detect large effect sizes. However, another possibility is that associations between conversational turns and vocabulary growth may be weaker in preterm children due to atypical neurodevelopment. The presence of the association in bilingual children may also indicate that cognitive advantages (e.g., Baralt and Mahoney 2020; Gillenson et al. 2023), heightened sensitivity to the communicative context and/or to interaction partners (e.g., Brojde et al. 2012; Gampe et al. 2019; Wermelinger et al. 2017; Yow and Markman 2016) associated with bilingualism may have served as protective factors. A limitation of the current study was the lack of a control group of typically developing children, which would have allowed us to make more direct comparisons between preterm and neurotypical language trajectories, particularly in bilingual contexts. Future research should incorporate groups of preterm and typically developing children to better understand the effects of bilingualism and prematurity on children’s language outcomes.

Children’s language outcomes were not the focus of the longitudinal project, and therefore, the study protocol did not collect sufficient information to determine whether a child had a language delay. We observed substantial growth in children’s vocabulary scores from 24 mo to 36 mo, bringing the average into the normal range. However, several children primarily produced 1–2-word utterances at 36 mo during parent–child interactions, which may be considered atypical. Nevertheless, it is challenging to ascertain whether any of the children in this study had a language delay. Considering the elevated risk for DLD in preterm children, it is likely that some of the children may have had language delays. Future work focusing specifically on language outcomes would be necessary to consider the relationship between the variables considered in the present study and a formal diagnosis of language difficulties or delays in bilingual children born preterm. There is some evidence to suggest that interventions targeting increased parental responsiveness and the use of expansions (where parents reformulate or elaborate on their child’s utterances) can be effective in enhancing the language outcomes for children with language delays (e.g., DeVeney et al. 2017; Zuccarini et al. 2020).

In conclusion, the present study contributes to the small body of work examining language development in monolingual and bilingual children born preterm. Our findings suggest that, despite differences in caregiver input, bilingual and monolingual preterm children develop comparable vocabulary skills and sentence complexity from 16 m to 36 m. These findings, although exploratory, underscore the robustness of bilingual language acquisition in children born prematurely. Future longitudinal studies with larger sample sizes of preterm children are needed to better understand the long-term effects of bilingualism on language development in children with neurodevelopmental vulnerabilities.

## Figures and Tables

**Table 1.** Participant characteristics.

	Monolingual (N = 9)	Bilingual (N = 9)
**Sex**		
Female	4 (44.4%)	4 (44.4%)
Male	5 (55.6%)	5 (55.6%)
**Gestational age at birth**		
Mean (SD)	30.48 (3.40)	30.91 (2.53)
Median [Min, Max]	31.71 [25.43, 34.14]	31.14 [27.22, 34.57]
**APGAR score at 1 min**		
Mean (SD)	6.44 (1.51)	5.00 (2.35)
Median [Min, Max]	7.00 [4.00, 8.00]	5.00 [1.00, 8.00]
**APGAR score at 5 min**		
Mean (SD)	7.78 (1.20)	7.44 (1.51)
Median [Min, Max]	8.00 [6.00, 9.00]	7.00 [6.00, 10.00]
**Mother’s years of education**		
Mean (SD)	13.67 (2.45)	13.11 (3.02)
Median [Min, Max]	12.00 [11.00, 18.00]	12.00 [8.00, 18.00]
**Mother’s Peabody Picture**		
**Vocabulary Test Standard Score**		
Mean (SD)	95.22 (11.52)	94.00 (13.41)
Median [Min, Max]	99.00 [76.00, 106.00]	96.00 [76.00, 111.00]
**Bayley Mental Development Index at 16 m**		
Mean (SD)	89.11 (13.86)	84.44 (10.85)
Median [Min, Max]	90.00 [64.00, 104.00]	84.00 [64.00, 98.00]
**Stanford-Binet Abbreviated IQ at 36 m**		
Mean (SD)	93.67 (12.59)	95.71 (17.65)
Median [Min, Max]	94.00 [76.00, 118.00]	100.00 [61.00, 109.00]

**Table 2.** Descriptives statistics for vocabulary and parent–child interaction variables.

	Monolingual (N = 9)	Bilingual (N = 9)
**Vocabulary at 16 m**		
Mean (SD)	32.33 (33.09)	20.00 (20.37)
Median [Min, Max]	24.50 [2.00, 97.00]	7.50 [3.00, 48.00]
**Vocabulary at 24 m**		
Mean (SD)	126.89 (112.40)	88.22 (77.39)
Median [Min, Max]	56.00 [13.00, 298.00]	51.00 [5.00, 242.00]
**Vocabulary at 36 m**		
Mean (SD)	285.56 (36.56)	264.00 (36.11)
Median [Min, Max]	302.00 [206.00, 310.00]	263.00 [219.00, 316.00]
**Child’s MLU at 16 m visit** ^a^		
Mean (SD)	0.15 (0.20)	0.09 (0.13)
Median [Min, Max]	0.08 [0.00, 0.57]	0.03 [0.00, 0.39]
**Child’s MLU at 36 m visit** ^a^		
Mean (SD)	2.22 (0.81)	1.56 (0.61)
Median [Min, Max]	2.31 [1.01, 3.40]	1.40 [0.99, 2.75]
**Mother’s NTW at 16 m visit** ^b^		
Mean (SD)	1093.89 (198.18)	733.56 (276.04)
Median [Min, Max]	1168.00 [760.00, 1308.00]	692.00 [201.00, 1149.00]
**Mother’s NTW at 36 m visit** ^b^		
Mean (SD)	1154.57 (327.90)	826.67 (321.82)
Median [Min, Max]	986.00 [830.00, 1591.00]	669.50 [527.00, 1294.00]
**Number of conversational turns at 16 m visit**		
Mean (SD)	45.33 (23.31)	51.56 (28.43)
Median [Min, Max]	43.00 [14.00, 80.00]	56.00 [19.00, 86.00]

a Mean length utterance in words.

b Number total words.

**Table 3.** Correlations for the entire sample.

	Child MLUw 16 m	Mother NTW 16 m	Conv. Turns 16 m	Child MLUw 36 m	Mother NTW 36 m	Vocab. 16 m	Vocab. 24 m
Mother NTW 16 m	−0.08						
Conversational Turns 16 m	0.39	−0.12					
Child MLUw 36 m	0.51	0.32	0.25				
Mother NTW 36 m	0.12	0.79 **	0.03	−0.01			
Vocabulary 16 m	0.64 **	0.14	0.09	0.49	0.61		
Vocabulary 24 m	0.62 *	0.11	0.66 *	0.71 **	0.15	0.42	
Vocabulary 36 m	0.24	0.23	0.03	0.73 *	−0.13	0.34	0.39

* *p* < 0.05;

** *p* < 0.01.

**Table 4.** Correlations for monolinguals.

	Child MLUw 16 m	Mother NTW 16 m	Conv. Turns 16 m	Child MLUw 36 m	Mother NTW 36 m	Vocab. 16 m	Vocab. 24 m
Mother NTW 16 m	−0.20						
Conversational Turns 16 m	0.42	−0.33					
Child MLUw 36 m	0.25	0.07	−0.21				
Mother NTW 36 m	0.05	0.59	−0.02	−0.34			
Vocabulary 16 m	0.66 *	0.66	0.83	0	0.6		
Vocabulary 24 m	0.63	−0.17	0.57	0.61	−0.14	0.54	
Vocabulary 36 m	0.26	0.17	−0.18	0.45	−0.38	0.03	0.23

* *p* < 0.05.

**Table 5.** Correlations for bilinguals.

	Child MLUw 16 m	Mother NTW 16 m	Conv. Turns 16 m	Child MLUw 36 m	Mother NTW 36 m	Vocab. 16 m	Vocab. 24 m
Mother NTW 16 m	−0.33						
Conversational Turns 16 m	0.29	0.38					
Child MLUw 36 m	0.30	0.20	0.66				
Mother NTW 36 m	−0.54	0.83	−0.14	−0.26			
Vocabulary 16 m	0.47	−0.37	−0.29	0.15	−0.05		
Vocabulary 24 m	0.46	0.29	0.79 **	0.75 **	−0.2	0.13	
Vocabulary 36 m	−0.07	−0.18	0.18	0.3	−0.6	0.49	0.29

** *p* < 0.01.

## Data Availability

The raw data and scripts supporting the conclusions of this article will be made available by the authors upon request.
